# 742 Is There a Mortality Benefit of Being Well-Insured in Burns?

**DOI:** 10.1093/jbcr/irac012.295

**Published:** 2022-03-23

**Authors:** Sanja Sljivic, Lori Chrisco, Rabia Nizamani, Booker King, Felicia Williams

**Affiliations:** University of North Carolina - Chapel Hill, Chapel Hill, North Carolina; North Carolina Jaycee Burn Center, Chapel Hill, North Carolina; UNC Jaycee Burn Center, Chapel Hill, North Carolina; UNC Jaycee Burn Center, Chapel Hill, North Carolina; UNC Jaycee Burn Center, Chapel Hill, North Carolina

## Abstract

**Introduction:**

The purpose of the Affordable Care Act was to make affordable health insurance available to more people, to cover adults with fewer resources, and to facilitate delivering health care in a cost-effective way. Burn care is both financially and medically intense, expensive, and prolonged. We aimed to compare outcomes of patients treated at a tertiary care center with no insurance, those considered under-insured (Medicare/Medicaid), and those with private/commercial insurance.

**Methods:**

Patients were identified using our institutional Burn Center registry and linked to the clinical and administrative data. All adult patients admitted to the Burn Center between January 1, 2011 and December 31, 2020 were eligible for inclusion. Demographics, length of stay (LOS), co-morbid conditions and mortality were evaluated. Statistical analysis was performed with Students’ t-test and chi-squared.

**Results:**

A total of 9,306 patients were admitted during the study period. Forty-one percent of patients had private/commercial insurance. Thirty-four percent were under-insured, while 25% of patients had no insurance. Total body surface area (TBSA) of the burn was significantly higher for the under-insured, p< 0.05. Mortality was significantly higher for the under-insured, p< 0.05. The average LOS for the under-insured was 14.7 days, which was significantly longer than that for the insured (9.2 days) and for those without insurance (7.4 days), p< 0.05.

**Conclusions:**

There are outcome disparities secondary to insurance coverage in burns. Under-insured patients had poorer outcomes than those with private/commercial insurance and those without insurance.

